# Anti-microbial Activity of Aqueous *Quercus infectoria* Gall Extract against Pathogenic *Leptospira*

**DOI:** 10.21315/mjms2018.25.4.4

**Published:** 2018-08-30

**Authors:** Husna Mustafa, Nabilah Ismail, Wan Nor Amilah Wan Abdul Wahab

**Affiliations:** 1 School of Health Sciences, Health Campus, Universiti Sains Malaysia, 16150 Kubang Kerian, Kelantan, Malaysia; 2 Department of Medical Microbiology and Parasitology, School of Medical Sciences, Health Campus, Universiti Sains Malaysia, 16150 Kubang Kerian, Kelantan, Malaysia

**Keywords:** Quercus infectoria gall extract, pathogenic Leptospira, anti-microbial activity, cell morphology, scanning electron microscope (SEM)

## Abstract

**Background:**

*Quercus infectoria* gall extract is known to have broad spectrum anti-microbial activity *in vitro*. This study was conducted to determine the anti-microbial activity of *Q. infectoria* gall extract against pathogenic *Leptospira* and to evaluate the morphological changes of extract-treated cells using a scanning electron microscope (SEM).

**Methods:**

A two-fold serial microdilution broth assay was used to determine the minimum inhibitory concentration (MIC) of aqueous *Q. infectoria* gall extract against the *L. interrogans* serovar Javanica and the *L. interrogans* serovar Icterohaemorrhagiae, at concentrations ranging from 4.00 mg/mL to 0.0078 mg/mL. The minimum bactericidal concentration (MBC) was determined by sub-culturing the broth from the microtiter plate wells that showed no apparent growth or turbidity to the freshly prepared Ellinghausen-McCullough-Johnson-Harris (EMJH) broth media, and it was subsequently observed under a dark field microscope following three weeks of incubation for purposes of growth detection. The cell morphology of both extract-treated and untreated *L. interrogans* serovar Icterohaemorhagiae was analysed using the SEM.

**Results:**

The results of the broth microdilution assay demonstrate that the aqueous *Q. infectoria* gall extract possesses anti-microbial activity against both of the *L. interrogans* serovars with MIC values of 0.125 mg/mL. The MBC values for the *L. interrogans* serovar Javanica and the *L. interrogans* serovar Icterohaemorhagiae are 0.125 mg/mL and 0.250 mg/mL, respectively. The SEM micrograph shows changes in shape and size of the extract-treated cells (at 8× MIC) in comparison to the untreated cells.

**Conclusion:**

The *Q. infectoria* gall extract displays anti-microbial inhibition and killing activity against the pathogenic *Leptospira* isolates, and thus has the potential for further exploration of its efficacy and use in the treatment of leptospirosis.

## Introduction

Leptospirosis is an infectious disease affecting animals and humans worldwide that is caused by the spiral-shaped bacteria known as the *Leptospira* species (1). Leptospirosis is reported to cause more than 500,000 cases of infection annually worldwide (2). Tropical and sub-tropical countries are ideal environments for the *Leptospira* to survive due to their high levels of humidity and warm temperatures (3). Currently, the data available for Malaysia are based on the Annual Report of Morbidity and Mortality from the Ministry of Health Hospitals, and the number of reported cases between the years 2004 and 2015 has increased from 263–5,370 (4). The disease is usually recognised only at its severe stage. During its early stages, the broad-spectrum symptoms of leptospirosis are often confused with other common bacterial infections (5). Thus, the antibiotic treatment administration is delayed for leptospirosis, which leads to a progressive and severe infection. Penicillin and doxycycline are used in the treatment, with intravenous penicillin G being mainly indicated for the severe form of leptospirosis. However, there is no conclusive evidence to show that penicillin or other antibiotics are effective in the prevention of complications and death arising from a severe infection (6).

Herbal alternatives to antibiotics for the treatment of infectious diseases are becoming more popular in our research culture and their promising treatment outcomes can be identified in the years to come. In addition, herbs may also be of great assistance in the prevention and control of certain infections. *Quercus infectoria* gall is one such herbal alternative and its use in traditional medicine for centuries is well known. There are several reports on the anti-microbial activity of the *Q. infectoria* gall extract against bacteria and yeast (7, 8). This extract is reported effective against multi-drug resistant (MDR) bacteria (8). A previous study indicates that there are several chemical components present in *Q. infectoria* gall, such as carbohydrates, lipids, mucilage, saponins and tannins (9). Another study reports that the tannins obtained from the gall serve as a natural defence against microbial infections and also have anti-inflammatory effects (10, 11). Tannins can be further classified into the hydrolysable and the condensed tannins. The *Q. infectoria* gall extract contains large amounts of tannic acid, which is considered to be a bioactive compound that leads to anti-microbial activity (12). Previously, pyrogallol (a type of hydrolysable tannin) is reported to be a major compound found in *Q. infectoria* galls (13).

Another plant extract that contains tannic acid, the *Phyllunthus amarus* plant extract, was tested positive for anti-leptospiral activity (14). However, the inhibition of the *Leptospira* spp. by a medicinal plant and its killing mechanisms are still poorly understood. The anti-leptospiral activity of the *Q. infectoria* galls is literally unknown. Based on its phytochemical substances, the gall extract might also possess anti-infective activity against pathogenic *Leptospira*. Therefore, this study was preliminarily conducted to determine the effects of the aqueous *Q. infectoria* gall extract against the *L. interrogans* serovars and to study the morphological changes of the *Leptospira* cells treated with the extract using a scanning electron microscope (SEM).

## Materials and Methods

### Preparation of the Q. infectoria Gall Extract

The *Q. infectoria* gall used in this study was obtained from Nur Saeida et al. (13) and its identification was previously performed based on its physical appearance and phytochemical composition (13, 15). The gall was first crushed in the mortar to small pieces and then grinded into a powder using an electrical grinder.

The extract was prepared by immersing the gall powder into a jar containing sterile distilled water with the weight to volume ratio of 1:5, which was then incubated at 50 °C for 72 h. Subsequently, the mixture was filtered using a coffee filter to separate the undissolved powder from the filtrate, and subsequent filtration was performed using the Whatmann filter paper. The filtrate product was then concentrated under reduced pressure at 80 °C using a rotary evaporator. The concentrated product was freeze-dried at −50 °C under a vacuum until a fine crystal-like crude extract was obtained, which was then stored in an air-tight container at 4 °C until further use.

### Preparation of the Leptospira Inoculum

The *L. interrogans* serovar Javanica and the *L. interrogans* serovar Icterohaemorrhagiae were obtained from the Department of Medical Microbiology and Parasitology, School of Medical Sciences, Universiti Sains Malaysia (USM). The inoculum was prepared by diluting a seven-day old *Leptospira* culture with fresh Ellinghausen-McCullough-Johnson-Harris (EMJH) broth to reach a bacterial density of 2 × 10^6^ CFU/mL by using a spectrophotometer at the 420 nm wavelength. The optical density (OD) of 0.32 at 420 nm approximately corresponded to 1 × 10^8^ CFU/mL (16). The OD of 0.064 was approximately equal to 2 × 10^7^ CFU/mL, and a 10-fold dilution was performed to reach a leptospiral density of 2 × 10^6^ CFU/mL.

### Determination of the Minimum Inhibitory Concentration (MIC) and the Minimum Bactericidal Concentration (MBC)

The extract’s MICs against both serovars were determined through a two-fold serial microdilution technique using 96 well-microplates (17), with extract concentrations ranging from 4.00 to 0.0078 mg/mL. Equal volumes of diluted inoculum suspensions (100 μL) were added to the designated test and control wells. The control wells were a positive (EMJH broth mixed with the organisms) and a negative (EMJH broth mixed with the extract) control groups. The test was performed in triplicates and the microplates were incubated at 30 °C for three days. The lowest concentration of the extract in the well-microplates that showed no turbidity after three days of incubation was recorded as the value of the MIC.

The MBC was determined by transferring 10 μL of broth from the wells without turbidity into a 15 mL falcon tube filled with 2 mL of a fresh EMJH broth medium. Then, the tubes were further incubated at 30 °C for three weeks. The MBC was documented if the lowest anti-microbial concentration yielded no growth of the *L. interrogans* serovars through observation under a dark field microscope.

### Morphological Analysis Using a Scanning Electron Microscope (SEM)

The cell morphology of the extract-treated *L. interrogans* serovar Icterohaemorrhagiae was studied under the SEM and compared with the negative and positive controls. Following incubation at 30 °C for 24 h, 2 mL of both treated (extract and penicillin-treated) and untreated (negative control) cultures were centrifuged at 45,000 rpm for 10 min to observe the presence of cells indicated by pellet formation at the bottom of the tube, and the supernatant was discarded. The tube was fixed using the McDowel Trump fixative at 4 °C for two hours. Afterwards, the sample was washed using 0.1 M phosphate buffer saline (PBS) by centrifugation at 42,000 rpm for 10 min. The supernatant was discarded, and the washing process was repeated two more times.

Subsequently, the sample was post-fixed with 1% osmium tetroxide at 4 °C for one hour and dehydrated using an ascending concentration of acetone (50%, 75%, 95% and 100%). After each addition of different acetone concentrations, the sample was centrifuged at 42,000 rpm for 10 min and the supernatant was discarded. At 100% acetone, the sample was dehydrated two more times before 100% hexamethyldisilazane (HDMS) was added. The sample was dried using a critical point dryer, later mounted on the SEM sample carbon stub and gold coated. The mounted slide was viewed under the SEM and a micrograph of the *Leptospira* cells was taken with an accelerating voltage of 5.00 kV.

## Results

### MIC and MBC Determination

**Figure 1:**
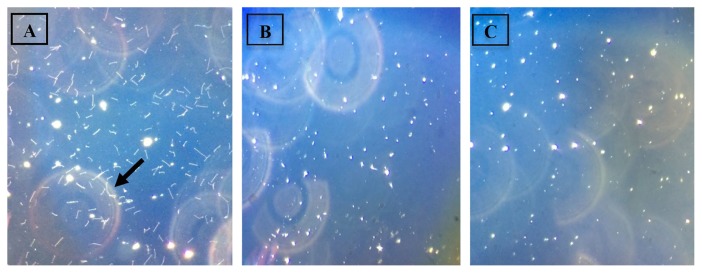
MBC determination of aqueous *Q. infectoria* gall extract against the *L. interrogans* serovars. These are the micrographs of the *L. interrogans* serovar Javanica cultures examined under the dark-field microscope (at 400× magnification) following three weeks incubation. A: The positive control (inoculum and broth) well shows a positive leptospiral growth indicated by the small white thread-like structures (arrow). B: The negative control (extract and broth) well shows the absence of growth or any intact cellular structure. C: Test well with MIC (0.125 mg/mL) shows the absence of growth or any intact cellular structure

**Figure 2:**
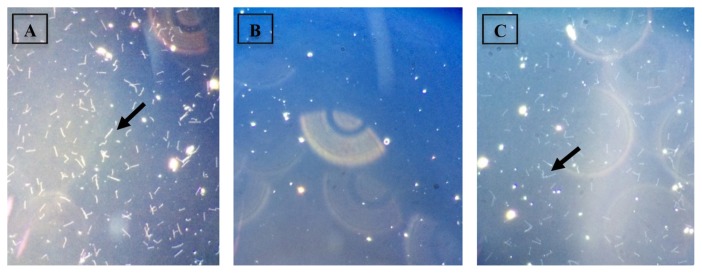
MBC determination of aqueous *Q. infectoria* gall extract against the *L. interrogans* serovars. These are the micrographs of the *L. interrogans* serovar Icterohaemorrhagiae cultures examined under the dark-field microscope (at 400× magnification) following three weeks incubation. A: The positive control (inoculum and broth) well shows a positive leptospiral growth indicated by the small white thread-like structures (arrow). B: The negative control (extract and broth) well shows the absence of growth or any intact cellular structure. C: Test well with MIC (0.125 mg/mL) shows the presence of leptospiral growth (arrow)

The MIC values of the aqueous *Q. infectoria* gall extract against both the *L. interrogans* serovar Javanica and the *L. interrogans* serovar Icterohaemorrhagiae were similar at 0.125 mg/mL, as shown in Table 1. The MBC for the *L. interrogans* serovar Javanica was similar to its MIC value, while the MBC value of the *L. interrogans* serovar Icterohaemorrhagiae was higher than its MIC value (Tables 2 and 3). The growth of the *L. interrogans* serovars was examined for MBC determination following three weeks of incubation, which was indicated by the absence or presence of motile spirochetes under the dark field microscope, as shown in Figures 1 and 2, respectively.

### Morphological Changes of the Treated Leptospira under the SEM

**Figure 3:**
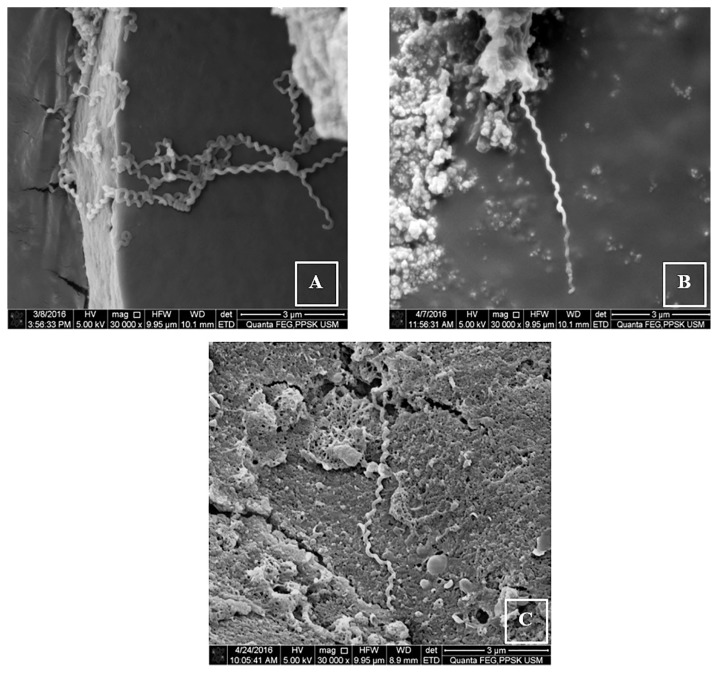
Scanning electron micrographs of the treated and untreated *L. interrogans* serovar Icterohaemorrhagiae cultures observed at 30,000× magnification. A: The negative control (untreated) smear shows the spiral-shaped structures of cells. B: The positive control (penicillin-treated) smear shows an elongated cell lacking of the helical shape. C: The extract-treated (at 8× MIC) smear shows a slightly elongated and deformed spiral-shaped cell

**Figure 4:**
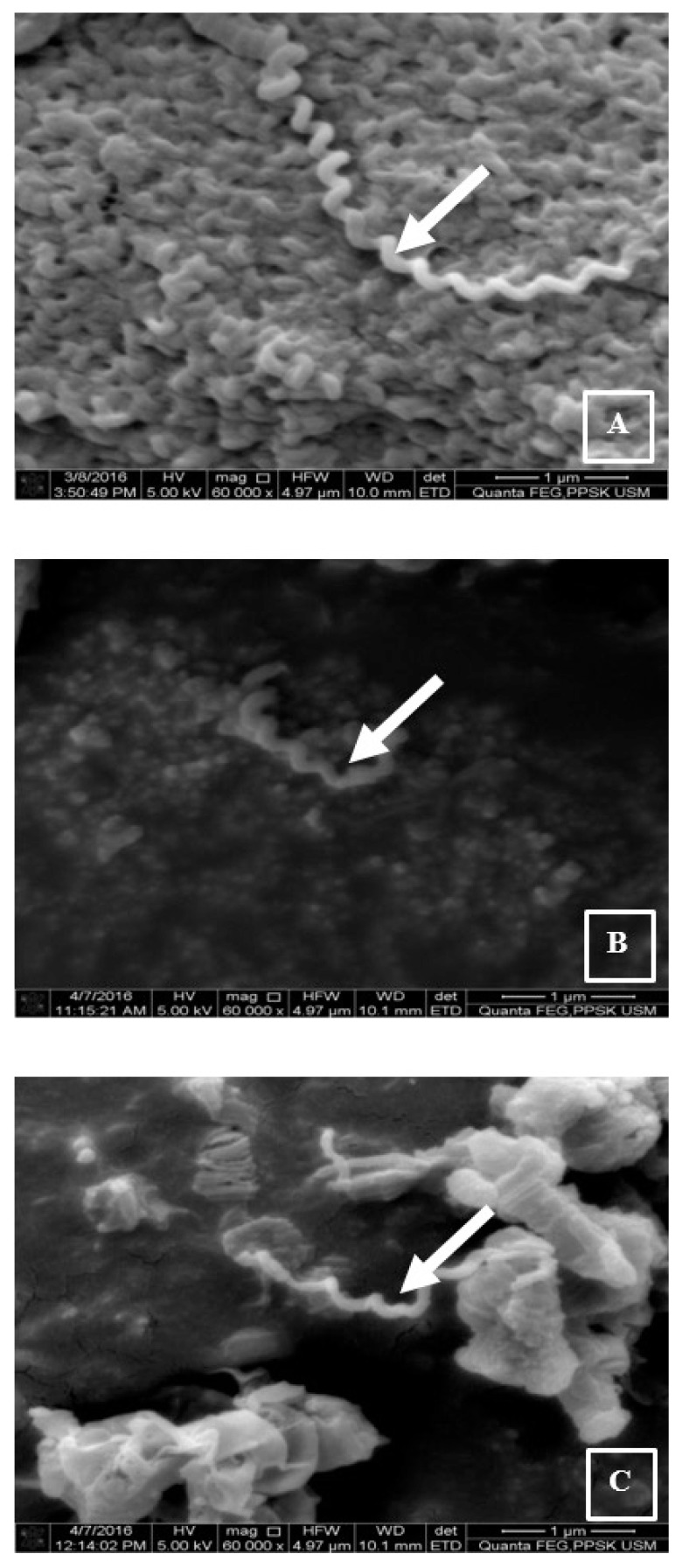
Scanning electron micrographs of the treated and untreated *L. interrogans* serovar Icterohaemorrhagiae cultures observed at 60,000× magnification. A: Negative control (untreated cell). B and C: Positive control (penicillin-treated) and extract-treated (at 8× MIC) smears show a relatively small size of cells in comparison to the one in the negative control (A)

The morphology of the *Leptospira* during the logarithm growth phase was observed under the SEM. The untreated cells were spiral and helical in shape, with a smooth surface (Figure 3A). The organisms were clumped together, and a hook formation could not be observed. The exposure of the bacterial cells to the extract at 8× MIC (2 mg/mL) showed a slight deformation in the cell shape with mild elongation (Figure 3C). The positive control group showed elongated cells lacking a helical shape (Figure 3B). In addition to the affected shape of the bacterial cells, the size of the extract-treated (Figure 4C) and penicillin-treated cells (Figure 4B) was relatively smaller and shorter in comparison to the untreated cells (Figure 4A), as observed under the SEM at 60,000× magnification.

## Discussion

Currently, there is no standard method for assessing anti-microbial agents for anti-leptospiral activity. The broth microdilution technique (17) is used in our study to determine the anti-microbial activity of the aqueous *Q. infectoria* gall extract against the pathogenic *Leptospira* serogroups. The inhibition activities of the aqueous *Q. infectoria* gall extract against the *L. interrogans* serovars are observed after three days incubation at 30 °C, as is indicated through observation of the well without turbidity on the 96-well-microplate. In this study water is selected as a solvent, since water is a polar and universal solvent that is most commonly used to extract plant products with anti-microbial activity (18). Furthermore, another study reports that the water extract of a plant contains a high concentration of both gallic and tannic acids in comparison to other types of solvents (19). This study also uses the same plant source as the one by Nur Saeida et al. (13), which identifies pyrogallic acid (1,2,3–benzenetriol) as a major compound of its extraction. Pyrogallol, a hydrolysable tannin with three hydroxyl groups and alpha-beta double bonds, reportedly plays an important role for anti-bacterial activity (13).

The aqueous *Q. infectoria* gall extract displays significant inhibitory effect on the *Leptospira* serovars, which is of clinical significance. The MIC value (0.125 mg/mL) of the aqueous *Q. infectoria* gall extract against the *L. interrogans* serovars is slightly higher than the MIC value of the aqueous *Q. infectoria* gall extract tested against the methicillin resistant coagulase negative *Staphylococcus* (MRCoNS), the methicillin resistant *Staphylococcus aureus* (MRSA) and the *Candida* sp., which show MIC values of 0.08 mg/mL (8) and 0.06 mg/mL (7), respectively. The ratio of the MBC to the MIC for the aqueous *Q. infectoria* gall extract against the *L. interrogans* serovar Javanica and the *L. interrogans* serovar Icterohaemorrhagiae is found to be less than four. Hence, the gall extract affects both of the *L. interrogans* serovars through bactericidal activity, a finding that also proposes a possible concentration dependent killing of leptospires.

The presence of a hydrolysable tannin like pyrogallol, which crosses the cell wall polysaccharide network and proteins, is a likely mechanism responsible for the inhibitory or killing effects on the organism. In addition, the hydrolysed tannin can also prevent the adherence of the bacteria to the host cell, as they have a structure similar to the bacteria-binding receptors that are present in urinary tract cells (20). Thus, the effects seen in this study on the structural changes of the *L. interrogans* serovar Icterohaemorrhagiae may be the result of pyrogallol action on the outer membrane of the organism, which lead to an inhibition of its growth.

The morphological defects of the leptospires when exposed to the gall extract also support, to some extent, an explanation of the cell surface or membrane disruptive mechanisms. The role of penicillin against the *L. interrogans* involves the inhibition of peptidoglycan formation by binding to the transpeptidase (21–23). Peptidoglycan is the inner cell wall that contains the penicillin-binding proteins (PBPs) (24). Thus, the mechanisms of the anti-microbial activity of penicillin occur internally within the inner membrane of the *Leptospira*, which could not be clearly revealed here using the SEM. Therefore, ultrastructural analysis using a transmission electron microscope should be done in order to further study the killing mechanisms of penicillin and the extract against *Leptospira* sp.

## Conclusions

The *Q. infectoria* gall extract has anti-microbial inhibition and killing activity against the *Leptospira* isolates. The findings presented in this research represent important data obtained regarding the anti-leptospiral activity of the *Q. infectoria* gall extract. Therefore, additional studies are needed to further evaluate the gall extract efficacy when used in combination with the currently available antibiotics and to assess its pre-clinical therapeutic use in more depth.

## Figures and Tables

**Table 1:** Microbroth dilution assay of aqueous *Q. infectoria* gall extract against *L. interrogans* serovars

*Q. infectoria* gall extract concentration (mg/mL)	*Leptospira interrogans* serovars	

	Javanica	Icterohaemorrhagiae
4.00	−	−
2.00	−	−
1.00	−	−
0.50	−	−
0.25	−	−
0.125	−	−
0.063	+	+
0.031	+	+
0.016	+	+
0.0078	+	+
Positive control	+	+
Negative control	−	−

No growth (−). Growth (+). Positive control: bacterial suspensions and broth; Negative control: extract and broth

**Table 2:** MBC determination of aqueous *Q. infectoria* gall extract against *L. interrogans* serovars

*Q. infectoria* gall extract concentration (mg/mL)	*Leptospira interrogans* serovars	

	Javanica	Icterohaemorrhagiae
4.000	−	−
2.000	−	−
1.000	−	−
0.500	−	−
0.250	−	−
0.125	−	+
0.063	+	+
0.031	ND	ND
0.016	ND	ND
0.0078	ND	ND
Positive control	+	+
Negative control	−	−

No growth (−). Growth (+). Positive control: bacterial suspensions and broth. Negative control: extract and broth. ND: not done because the well was turbid indicating the presence of the organisms within the well

**Table 3:** MIC and MBC values of aqueous *Q. infectoria* gall extract against *L. interrogans* serovars

*L. interrogans* serovars	Anti-microbial activity of aqueous *Q. infectoria* gall extract		

	MIC (mg/mL)	MBC (mg/mL)	MBC/MIC Ratio
Javanica	0.125	0.125	1
Icterohaemorrhagiae	0.125	0.250	2
